# A Rosemary Extract Rich in Carnosic Acid Selectively Modulates Caecum Microbiota and Inhibits β-Glucosidase Activity, Altering Fiber and Short Chain Fatty Acids Fecal Excretion in Lean and Obese Female Rats

**DOI:** 10.1371/journal.pone.0094687

**Published:** 2014-04-14

**Authors:** María Romo-Vaquero, María-Victoria Selma, Mar Larrosa, María Obiol, Rocío García-Villalba, Rocío González-Barrio, Nicolas Issaly, John Flanagan, Marc Roller, Francisco A. Tomás-Barberán, María-Teresa García-Conesa

**Affiliations:** 1 Research Group on Quality, Safety and Bioactivity of Plant Foods, Department of Food Science and Technology, Centro de Edafología y Biología Aplicada del Segura (CEBAS)-Consejo Superior de Investigaciones Científicas (CSIC), Espinardo, Murcia, Spain; 2 Department of Human Nutrition and Food Science, Faculty of Veterinary Sciences, University of Murcia, Espinardo, Murcia, Spain,; 3 Naturex SL, Quart de Poblet, Valencia, Spain; 4 Naturex SA, Site d'AgroParc, Avignon, France; German Institute of Human Nutrition Potsdam-Rehbrücke, Germany

## Abstract

**Background:**

Carnosic acid (CA) and rosemary extracts (RE) show body-weight, energy metabolism and inflammation regulatory properties in animal models but the mechanisms are not yet understood. Gut microbiota plays an important role in the host metabolism and inflammatory status and is modulated by the diet. The aim of this research was to investigate whether a RE enriched in CA affected caecum microbiota composition and activity in a rat model of genetic obesity.

**Methods and Principal Findings:**

A RE (40% CA) was administered with the diet (0.5% w/w) to lean (*fa*/+) and obese (*fa*/*fa*) female Zucker rats for 64 days. Changes in the microbiota composition and β-glucosidase activity in the caecum and in the levels of macronutrients and short chain fatty acids (SCFA) in feces were examined. The RE increased the *Blautia coccoides* and *Bacteroides*/*Prevotella* groups and reduced the *Lactobacillus*/*Leuconostoc*/*Pediococccus* group in both types of animals. *Clostridium leptum* was significantly decreased and *Bifidobacterium* increased only in the lean rats. β-Glucosidase activity was significantly reduced and fecal fiber excretion increased in the two genotypes. The RE also increased the main SCFA excreted in the feces of the obese rats but decreased them in the lean rats reflecting important differences in the uptake and metabolism of these molecules between the two genotypes.

**Conclusions:**

Our results indicate that the consumption of a RE enriched in CA modifies microbiota composition and decreases β-glucosidase activity in the caecum of female Zucker rats while it increases fiber fecal elimination. These results may contribute to explain the body weight gain reducing effects of the RE. The mutated leptin receptor of the obese animals significantly affects the microbiota composition, the SCFA fecal excretion and the host response to the RE intake.

## Introduction

Obesity and metabolic syndrome are increasingly common worldwide disorders attributed to excessive consumption of energy-dense foods, sedentary habits and genetic background [1], [2]. Impaired energy metabolism and low-grade inflammation are common characteristics of these disorders. The gut microbiota is also a key contributor to the development of obesity and metabolic disorders since it can modulate host energy metabolism and inflammatory status [3], [4]. Intestinal microbiota is mostly composed of anaerobic microbes distributed in two predominant phyla: Firmicutes (*Ruminococcus*, *Clostridium*, *Eubacterium*, *Lactobacillus*) and Bacteroidetes (*Alistipes*, *Prevotella*, *Bacteroides*) with significant numbers of Actinobacteria (*Bifidobacterium*) and Proteobacteria members [5], [6]. The intestinal microbiota plays an important role in fermenting and converting non-digested nutrients (i.e. resistant starch, fiber) into nutrients to provide further energy [5], [7]. This ability is sustained by the production of an array of enzymes including glycoside hydrolases that catalyze the breakdown of carbohydrates to fermentable monosaccharides [6]. β-Glucosidases are a family of glucohydrolases that act upon β1→4 bonds and contributes to the hydrolysis and fermentation of cellulose. The main end products of fermentation by the microbiota are short-chain fatty acids (SCFA), typically acetate, propionate and butyrate which are largely absorbed and metabolized by the host organism. SCFA can act as signaling molecules and regulate energy metabolism and inflammatory responses in the host [8]. They also play important roles as substrates for glucose, cholesterol and lipid metabolism providing up to 10% of the daily caloric intake [9]. Microbial metabolism can also affect gut-hormone production and intestinal permeability causing elevated systemic levels of lipopolysaccharide which contributes to the low-grade systemic inflammation associated with obesity and metabolic syndrome [10].

Obesity is associated with changes in the abundance at the level of phylum, genus or species of gut microbiota. In mice, Firmicutes is increased and Bacteroidetes decreased in obese animals [3] but, in humans, differences between obese and normal-weight individuals as well as changes following weight reduction due to caloric-restriction are not consistent [5]. It does appear, however, that the mutual influence of gut flora composition and overall weight conditions is connected to differences in the energy-reabsorbing potential of different ratios of Firmicutes and Bacteroidetes, especially in the digestion of dietary fats and carbohydrates [11], [12]. Modulation of the gut microbiota through the diet to enhance host health and to reduce the incidence of obesity and associated disorders is an important line of research [13]. According to FAO, prebiotics are ‘non-viable food components that confer a health benefit on the host associated with modulation of the microbiota [14]. This concept has been thoroughly revised and since most studied prebiotics are fibers, the latest definition recognizes that prebiotics are ‘selectively fermented ingredients that promote the selective stimulation of growth and/or activities of one or a limited number of microbial genus(era)/species in the gut microbiota that confer(s) health benefits to the host’ [15], [16]. Plant extracts enriched in bioactive compounds are also widely investigated as an additional strategy to combat obesity and metabolic disorders since some of their components and (or) derived metabolites appear to exert a number of metabolic regulatory and anti-inflammatory properties as well as to modify the intestinal environment through modulation of the microbiota [17]. We have previously reported that a rosemary (*Rosmarinus officinalis*, Lamiaceae) extract (RE) enriched in the bioactive compound carnosic acid (CA) has body weight, serum lipids and insulin lowering effects in female Zucker rats, more noticeable in the lean animals. These effects were partially attributed to the inhibition of a pre-duodenal butyrate esterase activity and potential reduction of fat absorption [18]. We have also shown that the RE differentially modulates the production of anti- and pro-inflammatory cytokines as well as hepatic metabolic gene expression in the lean and obese animals [19] but, the mechanisms triggering these effects and the differences found between the two genotypes are not yet fully understood.

To gain further insight into the potential mechanisms by which the RE rich in CA may regulate energy metabolism and inflammatory status as well as to further understand the different response found between lean and obese Zucker rats [18], [19], we have examined changes in representative groups of Firmicutes, Bacteroidetes and Actinobacteria present in the caecum of obese (*fa/fa*) female Zucker rats and of their lean (*fa/+*) counterparts following diet supplementation with the RE for 64 days. In addition, we have measured and compared the effects of the RE on caecum β-glucosidase activity and on the content of water and macronutrients (fat, protein, ash and fiber) as well as SCFA in the fecal output between the two types of animals.

## Materials and Methods

### Materials

The RE used in this study (reference GAX00011 batch number A273/045/A10) was kindly provided by Naturex (Valencia, Spain) and was prepared according to a patented method [20]. The patented extract contains water (2.2%), fat (7.9%), protein (0.6%), ash (6.2%), carbohydrates (29.1%), total dietary fiber (1%), CA (38.9±1.7%), carnosol (6.5±0.1%) and CA 12-methyl ether (6.9±0.6%). Other phenolic compounds were detected at very small quantities and were not quantified (rosmarinic acid, rosmanol epirosmanol, epiisorosmanol, epiisorosmanol ethyl ether, rosmadial, caffeic acid hexoside, medioresinol, isorhamnetin 3-*O*-hexoside, homoplantagin, cirsimaritin and 4′-methoxytectochrysin) [21]. Porcine pancreatic α-amylase, soluble starch, 3,5-dinitrosalicylic acid (DNS), *p*-nitrophenyl-β-D-glucopyranoside, carnosic acid, carnosol, acetic acid (C2), propionic acid (C3), isobutyric acid (i-C4), butyric acid (C4), isovaleric acid (i-C5), valeric acid (C5), and 4-methyl valeric acid (internal standard) and *p*-nitrophenol were all purchased from Sigma-Aldrich (St. Louis, MO, USA) All other reagents were of chemical grade. Water was deionized by using a Milli-Q-system (Millipore, Bedford, MA, USA).

### Animals and Experimental Design

This study was carried out at the Experimental Animal Unit of the University of Murcia, Spain (Registration number: REGA ES 300305440012). All procedures were performed in strict accordance with the European Union regarding animal experimentation (Directive of the European Council 86/609/EC and Spanish RD 1201/2005). The protocols were approved by the Bioethics Committee of the Department of Agriculture, Fisheries and Livestock of the Autonomous Council of Government of Murcia, Spain (Permit Number: C1311031102).

This study is a continuation of two previously published studies, using the same animals and study protocol as previously described [18], [19]. Briefly, female Zucker lean (*fa*/+) (Le, n = 14) and obese (*fa*/*fa*) (Ob, n = 10) rats (105.5±13.3 g and 148.5±22.9 g, respectively) were fed with a standard diet (CT) or with the diet supplemented with RE (0.5% w/w) for 64 days. Full diets composition (major nutrients) is described in Table S1. Body weight, food intake and feces weight were recorded throughout the study. A total of 30 fecal stools per group of rats were scanned using and ImageScanner II (Amersham Bioscience, General Electric, Barcelona, Spain) and the Labscan v5.0 software. Images were analyzed with the Microm Image Processing software (MIP 4.5, CID, Barcelona, Spain) and the maximum and minimum diameters were measured. The volume (V) of the stools was estimated using the formula for an ellipsoid V = 4/3×π×a×b×c (a = maximun diameter/2; b = c = minimum diameter/2). At the end of the study (rats ∼15-weeks old), non-fasted animals were anaesthetized by injection with a mixture (1∶1, v/v; 1 mL/kg of body weight) of ketamine (Imalgene 1000; Merial Laboratories, Barcelona, Spain) and xylazine (Xilagesic 2%; Calier Laboratories, Barcelona, Spain) and sacrificed by heart puncture exsanguination. The caecum and its contents were collected and weighed (total weight). We removed the contents, washed the tissue thoroughly and weighed again (tissue weight). The weight of the contents was calculated by the difference between total and tissue weight. All the samples were frozen in liquid nitrogen and stored at −80°C until further analysis.

### Microbial analysis of the caecum contents

Quantitative PCR (qPCR) was performed to determine the effect of the supplementation with RE on total bacteria counts and on the composition of the caecum microbial community. Caecum content (∼20 mg) were added to 2 mL tubes containing specialized beads (MP Biomedicals, LLC, Ohio, USA) and homogenized in the lysing matrix using the FastPrep Instrument (30 s, speed setting of 5.5). DNA was then extracted using a commercial extraction kit (QIAampR DNA Stool Mini Kit,Qiagen Inc., Valencia,CA) following the manufacturer's instructions. qPCR was performed using an ABI 7500 Sequence Detection System (ABI, Applied Biosystems, Madrid, Spain). Metagenomic DNA from dominant groups of fecal bacteria was quantified by real-time qPCR with 16S rRNA species specific primers as indicated in Table S2. Amplification and detection were carried out in 96-well plates with TaqMan Universal PCR 2× Master Mix (ABI) or with SYBR-Green PCR 2× Master Mix (ABI). Each reaction was run in duplicate in a final volume of 25 µL with 0.20 µM final concentration of each primer, 0.25 µM final concentration of each probe and 10 µL of appropriate dilutions of DNA samples. Amplifications were carried out using the following conditions: 1 cycle at 95°C for 10 min, followed by 40 cycles of 95°C for 30 s, 60°C for 1 min. For SYBR-Green amplifications, a melting step was added to improve amplification specificity. Bacterial population in the caecum samples were quantified using standard curves made by serial dilution of known concentrations of genomic DNA isolated from: *Escherichia coli* CECT 515^T^ for total bacteria [22]; *Bifidobacterium longum* DSM 20088^T^ for *Bifidobacterium* genus; *Lactobacillus plantarum* CECT 748^T^ for the quantification of *Lactobacillus/Leuconostoc/Pediococcus* group; *Clostridium leptum* DSM 753^T^ for the *Clostridium leptum* group; *Blautia coccoides* DSM 935^T^ for the *Blautia coccoides/Eubacterium rectal*e group and *Bacteroides ovatus* DSM 1896^T^ for the *Bacteroides/Prevotella* group [23]–[25]. DNA from pure cultures was isolated by using the DNeasy Blood & Tissue Kit (Qiagen) following the manufacturer's instructions. Standard curves were prepared by plotting threshold cycles (Ct) *vs*. total number of bacteria (CFU). Cell counts before DNA extraction were determined by the standard plate count method. The caecum content samples CFU were extrapolated from the averaged standard curves (n = 3). Total bacteria are presented as the mean log_10_ value per g of caecum content (fresh weight). Results for each bacterial group are shown as relative abundance (%) of total bacteria.

### Fecal samples composition

The stools were rapidly frozen at −80°C and freeze-dried in a lyophilizer (Alpha 1–4; Martin Christ, Osterode, Germany) for 48 h. Fecal water content was calculated by the weight difference between fresh stools (weighed immediately after collection) and dried stools. The dried stools were then ground and used for the analysis of protein, fat, total dietary fiber and ash using the AOAC official methods, 955.04, 920.39C, 985.29, and 923.03, respectively [26].

### Short-chain fatty acids analysis

SCFA levels were quantified according to a previously published protocol [27]. Fecal samples were weighed and suspended in 1 mL of water with 0.5% orthophosphoric acid per 0.1 g of sample and frozen at −20°C. Once thawed, the fecal suspensions were homogenized with a vortex for about 2 min and centrifuged for 10 min at 17,949×*g*. Supernatants were extracted with 1 volume of ethyl acetate for 2 min and centrifuged for 10 min at 17,949×*g*. Organic extracts were stored at −20°C. Prior to analysis, a 600 µL volume of the organic phase was transferred into a tube and 4-methyl valeric acid added as internal standard (500 µM final concentration). Samples were directly injected onto the GC-MS column. The GC-MS system consisted of an Agilent 7890A (Agilent Technologies, Palo Alto, CA, USA), equipped with an automatic liquid sampler (MPS2) (Gerstel, Mülheim, Germany) and coupled to an Agilent 5975C mass selective detector. A fused silica capillary column DB-WAXetr (30 m, 0.25 mm id, 0.25 µm film thickness) and 1 mL/min of helium as carried gas were used for the separation. Samples (1 µL) were injected in splitless mode with an injector temperature of 250°C. The column temperature was initially 90°C, then increased to 150°C at 15°C/min, to 170°C at 5°C/min, and finally to 250°C at 20°C/min and kept at this temperature for 2 min (total time 14 min). Solvent delay was 3.5 min. The detector was operated in electron impact ionization mode (electron energy 70 eV), scanning the 30–250 m/z range. The temperature of the ion source, quadrupole, and interface were 230, 150, and 280°C, respectively. Identification of the SCFAs was based on the retention times of the standard compounds and with the assistance of the NIST 08 and Wiley7N libraries. A characteristic single ion was selected for the quantification of each compound: propionic acid 74, isobutyric acid 88, butyric acid 73, isovaleric acid 87 and valeric acid 73.

### Enzyme activity

#### α-Amylase activity

Small intestine content samples from control and RE-supplemented lean rats were prepared as previously described [18]. Pancreatic α-amylase was prepared in ultrapure water (∼1000 U/mL) immediately before use. Human saliva samples were collected (n = 3 subjects) using a plastic Pasteur pipette placed under the tongue for 30 s immediately before use. Volunteers did not eat or drink anything for at least 30 min and rinsed their mouth with water before saliva collection. Enzyme reaction (final volume: 1 mL) was carried out in 20 mM sodium phosphate buffer (6.7 mM NaCl, pH 6.9) containing 90 µL of the substrate (1% boiled starch solution) tested against: i) 50 µL of undiluted intestine content supernatant, ii) 10 U of pancreatic α-amylase, or iii) 50 µL of human saliva. The reaction took place at 37°C for 20 min and thereafter 100 µL of DNS reagent solution (32 mM DNS, 1 M sodium potassium tartrate in 0.4 M NaOH solution) were added and samples were boiled for 15 min. Samples were then cooled on ice, diluted 1∶2 with ultrapure water and absorbance recorded at 540 nm in an UV-VIS spectrophotometer (Jasco V-630, Tokyo, Japan).

#### β-Glucosidase activity

β-Glucosidase activity was determined in the caecum digesta by measuring the rate of *p*-nitrophenol release from *p*-nitrophenyl-β-D-glucopyranoside. Caecum samples (∼100 mg) were vortex mixed with 0.4 mL of 50 mM phosphate buffer (pH 6.5) and sonicated in ice for 15 min followed by centrifugation at 1699×*g* for 10 min. The reaction mixture contained 20 µL of appropriately diluted caecum supernatant in 50 mM phosphate buffer (pH 6.5) and 80 µL of substrate solution (2 mM) and was incubated for 30 min at 37°C. The reaction was stopped with 100 µL NaOH (0.5 M) and *p*-nitrophenol quantified at 405 nm in 96-well plates in a microplate reader (Tecan Infinite M200, Tecan, Grödig, Austria). Data were collected in triplicate. Total protein content in the gut content samples was measured by the DC colorimetric assay at 750 nm (BioRad, Barcelona, Spain) based on a bovine serum albumin standard curve.

CA (prepared in 4∶1 ACN:DMSO, final solvent concentration <10%) was added to the reaction mixtures to investigate the specific effect of the compound on the gut content samples. Enzyme activity was calculated as U/mg of protein.

### Statistical analysis

Results are shown as the mean value ± SD. Differences between groups were compared using an unpaired Student's *t* test. Results with a two-sided *P*-value <0.05 were considered statistically significant. Principal component analysis (PCA) was used to visualize the relationship between the caecum bacterial groups examined in the Zucker lean and obese rats fed the control diet or the diet supplemented with RE and the biochemical variables (triglycerides, cholesterol, insulin, leptin, adiponectin, tumor necrosis factor alpha (TNF-α), and interleukin 1 beta (IL-1β)) previously determined in the serum of the same animals [18], [19]. All statistical analyses were performed using SPSS 19.0 for Windows.

## Results

### Effects of RE supplementation on body and caecum weight

Final body weight for the lean CT rats was 229.6±14.6 g compared to 210.3±15.0 g for the RE-supplemented lean group (total body weight gain: 103.3 g *vs*. 80.1 g, respectively; *P*<0.01). In the obese group, the final body weights were 455.2±14.4 g for the CT rats and 409.2±31.4 for the RE-supplemented rats (total body weight gain: 270.2 g *vs*. 235.0 g, respectively; *P*<0.05). Total energy consumption was ∼1.7-fold higher in the obese rats than in the lean ones (∼75 *vs*. ∼44 kcal/rat day) while the relative food intake (RFI) was similar in the two genotypes and was not significantly affected by the supplementation with the RE (Figure S1).

**Figure 1:**
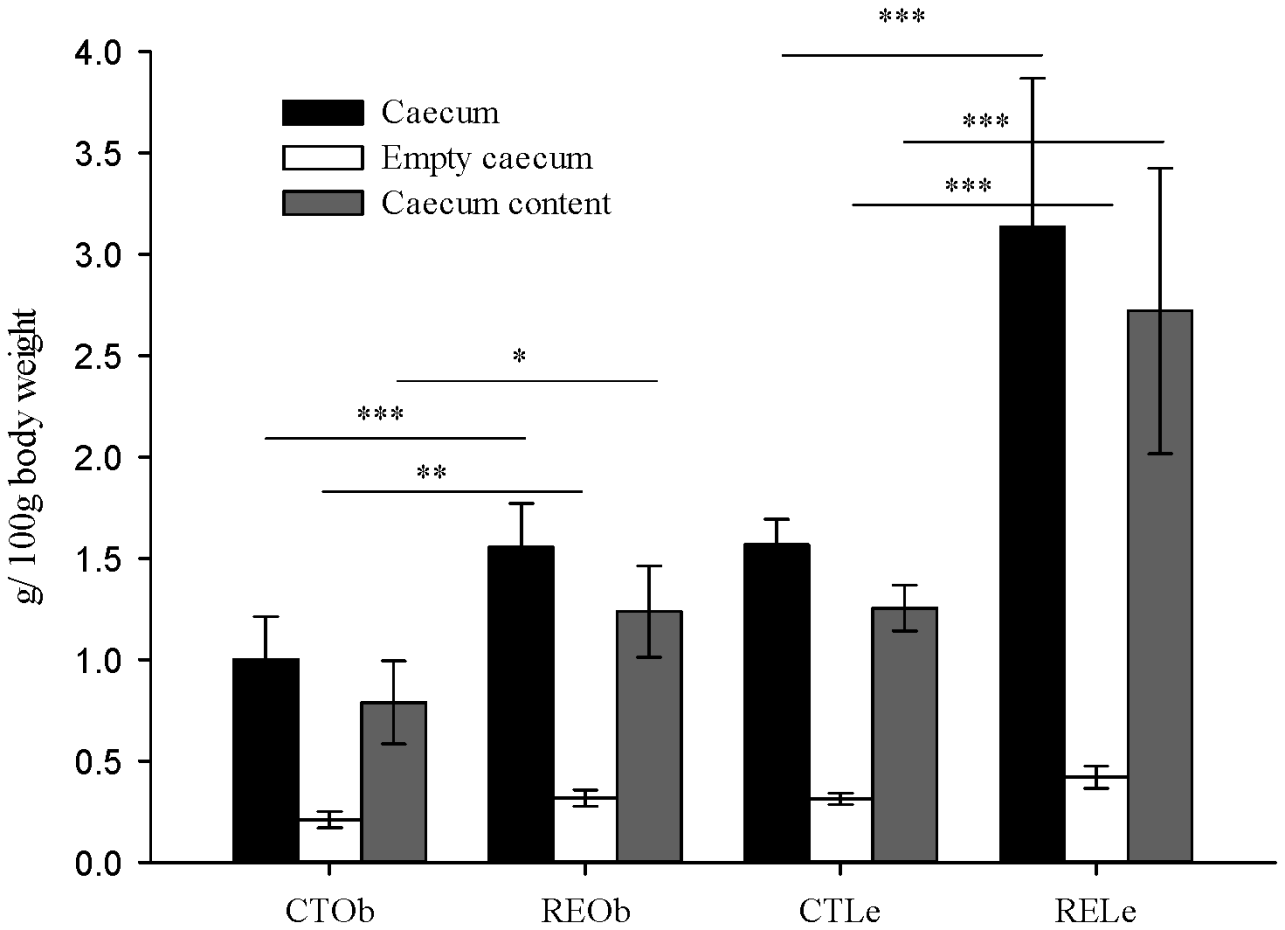
Effect of the RE-supplementation on full caecum, empty caecum and caecum content weight. Obese (Ob) and lean (Le) female Zucker rats were fed a control diet (CT) or the diet supplemented with the rosemary extract (RE) enriched in carnosic acid (CA ∼40%). Values are expressed as g/100 g of body weight. Bars indicate mean values ± SD (n = 7 lean animals, n = 5 obese animals per group). Significant differences between control and RE-supplemented groups are also indicated: *P<0.05, **P<0.01, ***P<0.001.

We found that supplementation with the RE significantly enlarged the size and weight of the caecum in lean and obese rats. Relative values (g/100 g body weight) for total weight, caecum content weight and organ weight are shown in Figure 1. Caecum total weight was increased from 3.54±0.23 g f.w. (fresh weight) in the control lean rats to 6.60±1.76 g f.w. (1.9-fold) in the rats supplemented with the RE. In the obese rats, the caecum with its contents weighed 4.55±0.99 g f.w. in the control group and 6.37±1.01 g f.w. (1.4-fold) in the RE-supplemented animals.

### Microbial population changes associated with the RE intake

**Figure 2:**
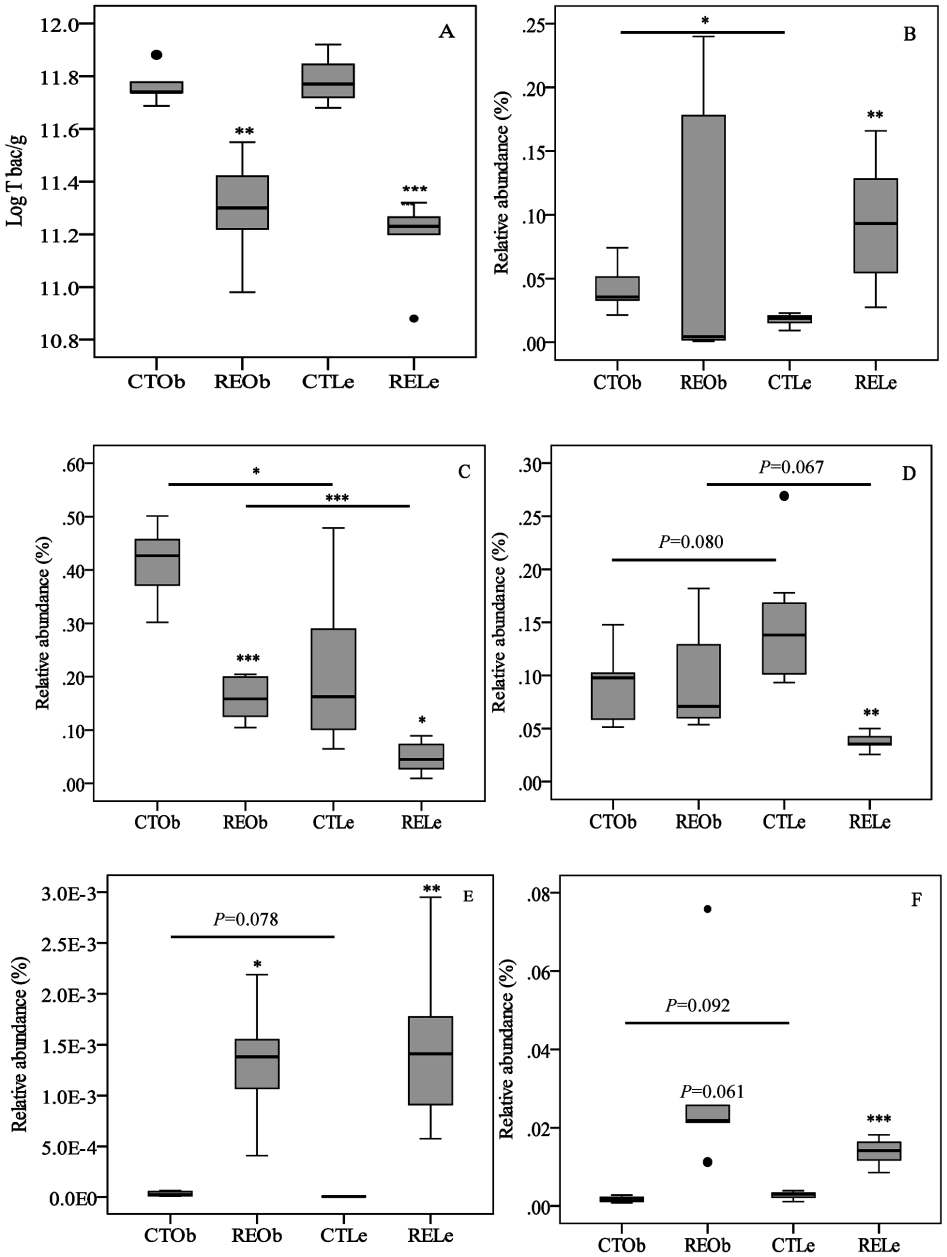
Box-and-Whisker plots of the content of bacteria in the caecum of obese (Ob) and lean (Le) female Zucker rats fed a control diet (CT) or the diet supplemented with a rosemary extract (RE) enriched in CA (40%). Caecum content of: A) Total bacteria (T bac) indicated as log_10_ of the value per gram of caecum content (fresh weight), B) *Bifidobacterium* genus, C) *Lactobacillus/Leuconostoc/Pediococcus* group, D) *Clostridium leptum* group, E) *Blautia coccoides* group and F) *Bacteroides-Prevotella* group. Results for each group are presented as relative abundance (%) of total bacteria. Horizontal lines represent the comparison between obese and lean rats. Significant differences are indicated by *P<0.05, **P<0.01, ***P<0.001. *P* values below 0.1are also displayed as indication of a trend.

qPCR analyses of the different groups of bacteria in the caecum content samples obtained from lean and obese control rats as well as from the RE-supplemented groups are shown in Figure 2 A–F. Comparison between the control lean (CTLe) and obese (CTOb) animals showed that the total numbers of bacteria were not significantly different between the two genotypes (Figure 2A). We detected, though, some significant differences between control lean (CTLe) and obese (CTOb) rats for the specific groups of bacteria examined: the relative abundance of the *Bifidobacterium* and of the *Lactobacillus/Leuconostoc/Pediococcus* groups was higher in the obese rats than in their lean counterparts (*P*<0.05) (Figure 2B and 2C, respectively). We also detected that the *C. leptum* and *Bacteroides*/*Prevotella* groups were lower (Figure 2D and 2F) and that the *B. coccoides* group was higher in the obese control rats than in the lean ones, although these results did not reach significance (*P*<0.1). Supplementation with the RE caused a significant reduction of total bacteria (Figure 2A) as well as of the *Lactobacillus/Leuconostoc/Pediococcus* group (Figure 2C) whereas the *B. coccoides* was significantly increased (Figure 2E), in both type of animals. The *Bacteroides/Prevotella* group was also increased after the RE intake in both genotypes, most significantly in the lean rats (Figure 2F). Notably, the *Bifidobacterium* and *C. leptum* groups were significantly increased and decreased, respectively, by the RE in the lean animals (Figure 2B and 2D).

**Figure 3:**
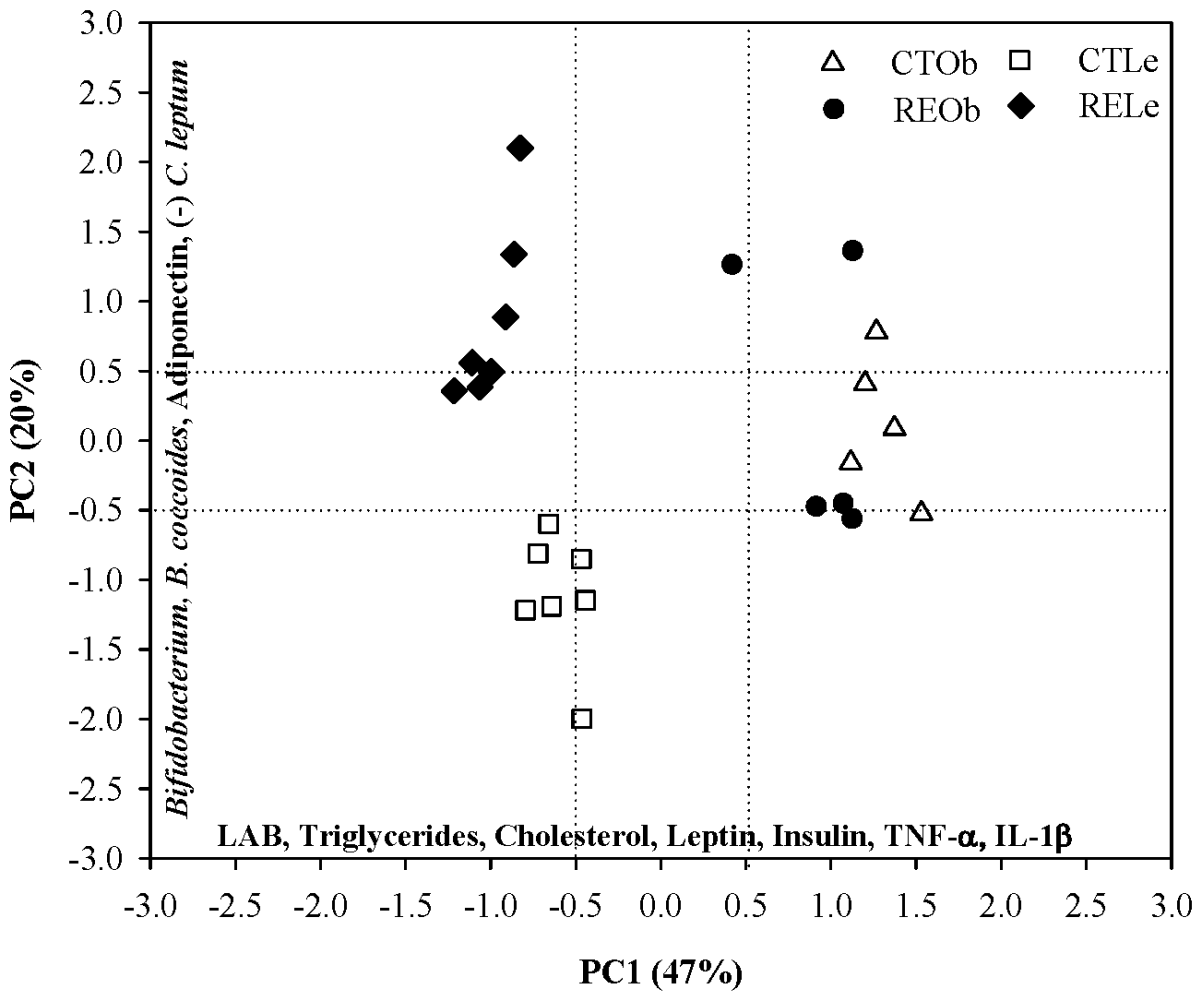
Discriminant principal component analysis (PCA). Analysis of the caecum bacterial groups in Zucker male obese (Ob) and lean (Le) rats and the serum triglycerides, cholesterol, leptin, insulin, adiponectin, TNF-α, and IL-1β previously measured in the same animals [16], [17]. Plot analysis of the four animal groups: control lean rats (CTLe, open squares), RE-supplemented lean rats (RELe, black diamonds), control obese rats (CTOb, open triangles) and RE-supplemented obese rats (REOb, black circles).

Discriminant PCA of the caecum bacterial groups in Zucker female obese and lean rats and the serum lipids, adipokines and cytokines previously measured in the same animals [18], [19] is shown in Figure 3. The analysis identified 2 components accounting for 67% of the total variability. The first principal component (PC1) explained 47% of the variability and was determined by most of the serum variables (triglycerides, cholesterol, leptin, insulin, TNF-α, and IL-1β) and by the *Lactobacillus/Leuconostoc/Pediococcus* group (LAB). The second component (PC2) explained 20% of the variability and was represented mostly by the *Bifidobacterium* genus, the *C. leptum* and *B. coccoides* groups, and adiponectin. The plot illustrates that, PC1 and PC2 clearly differentiate between the lean and the obese phenotypes (*P*≤0.001). PC1 and PC2 also discriminate between control and RE-supplemented lean rats (*P*≤0.001) but not between control and supplemented obese rats. Thus, the intake of the RE causes a marked different response between the lean and obese rats.

### Effects of the RE supplementation on enzyme activity

**Figure 4:**
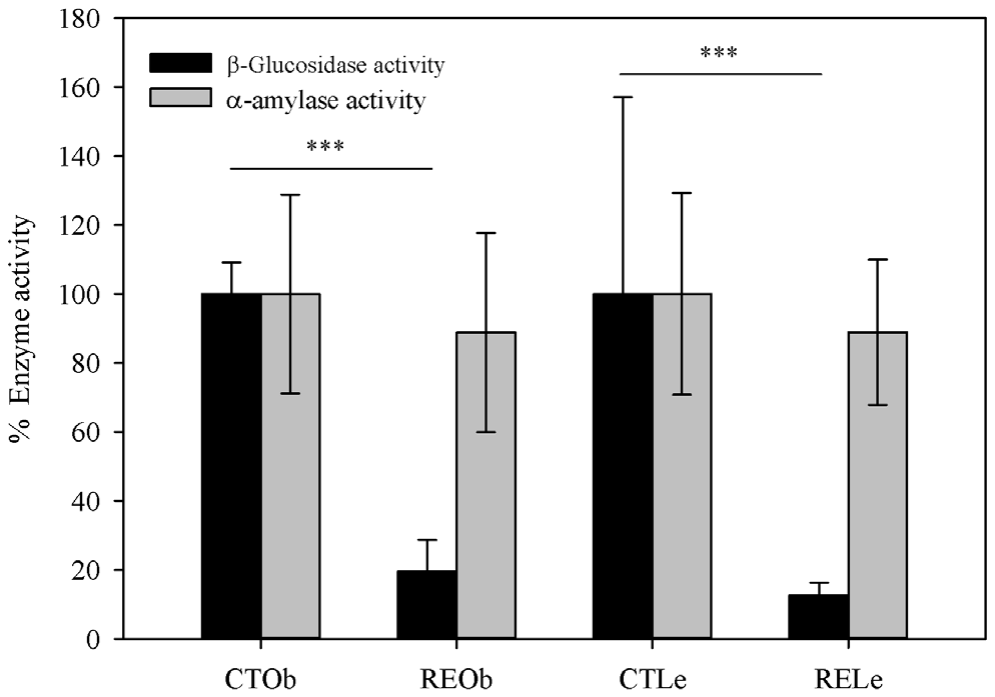
Effect of the RE-supplementation on gut enzyme activity. α-Amylase activity in the small intestine and β-glucosidase activity in the caecum of obese (Ob) and lean (Le) Zucker female rats. Results are shown as % of inhibition respect to the activity in the corresponding control (CT) group.

Under the conditions of our assay, CA inhibited porcine pancreatic α-amylase only at very high concentrations (∼83% inhibition at 100 µM CA and ∼52% inhibition at 360 µM CA) and did not show any effect on human salivary α-amylase at the same concentrations. α-Amylase activity was slightly, but not significantly, lowered in the small intestine of the animals that consumed the RE (Figure 4). Rats supplemented with the RE exhibited, however, a substantial reduction in caecum β-glucosidase activity (96.1%, *P* = 0.005 in the obese animals and 92.2%, *P* = 0.002 in the lean animals) (Figure 4). The addition of CA to the reaction mixtures (final concentrations ∼70–300 µM) did not have any effect on the α-amylase or β-glucosidase activities.

### Effects of RE supplementation on the fecal output

Total fecal weight (g) and fecal volume (mm^3^) were augmented during the course of the experiment in the lean and obese rats supplemented with the RE (Figure S2). Feces volume and weight values at the end of the study are included in Table 1. Analysis of the fecal macronutrients composition are also presented in Table 1 and provide evidence that the observed volume and weight increment was mostly associated with a significant increase in the water content (∼1.3-fold) and of total fiber (∼1.3–1.4-fold) in the feces of the animals fed the RE-supplemented diet.

**Table 1:** Estimated daily intake, total fecal output and percentage eliminated of main macronutrients in the obese (Ob) and lean (Le) Zucker rats. Control group (CT) and rosemary extract (RE)-supplemented rats.

	CTOb	REOb	CTLe	RELe
*Total daily intake (rat^−1^ day^−1^)* a				
Total energy (Kcal)	75.0±10.9	71.9±7.1	43.9±9.6	43.8±4.1
Water (g)	21.7±42.7	22.9±41.0	19.7±34.2	20.0±41.1
Fat (mg)	1030	950	610	580
Protein (mg)	3698	3517	2165	2140
Ashes (mg)	1215	1163	712	708
Fiber^b^ (mg)	5714	5462	3330	3308
Carbohydrates (mg)	12411	11836	7268	7203
*Fecal daily output*				
Fecal volume (mm^3^)	247±36	349±59**	234±31	288±45**
Fecal weight (g, f. w.)^c^	5.43±0.6	6.64±0.4*	2.77±0.2	3.64±0.3*
Fecal composition				
Water	60.1%^d^	61.8%	56.8%	58.3%
	3261±214^e^	4102±164*	1573±76	2123±196*
	(15.0%)^f^	(17.9%)	(7.9%)	(10.6%)
Fat	1.1%	0.9%	1.2%	1.0%
	57.8±2.7	58.0±1.6	32.0±1.5	35.5±7.2
	(5.6%)	(6.1%)	(5.2%)	(6.2%)
Protein	4.9%	4.5%	6.2%	4.4%
	264.3±1.6	295.4±1.1	171.9±1.5	160.0±1.6
	(7.2%)	(8.4%)	(7.9%)	(7.5%)
Ashes	5.6%	4.4%	5.5%	4.4%
	302.4±1.7	290.8±4.6	153.5±1.0	160.3±6.5
	(24.9%)	(25.0%)	(21.3%)	(22.6%)
Fiber	24.1%	24.7%	26.1%	28.0%
	1307±1.6	1638±1.1*	722±1.5	1022±1.6*
	(22.9%)	(30.0%)	(21.7%)	(30.1%)
Carbohydrates^g^	4.4%	3.9%	4.3%	3.9%
	264.4	295.5	171.7	159.8
	(2.13%)	(2.49%)	(2.36%)	(2.22%)

a : values expressed per rat and per day and calculated using the average daily diet intake and diet composition presented in Table S1; ^b^total dietary fiber (AOAC 985.29); ^c^: f.w., fresh weight; ^d^: g/100 fecal fresh weight; ^e^: mg/rat day; ^f^: (% eliminated with respect to daily intake); ^g^: carbohydrates were estimated by difference; *: *P*<0.05 and **: *P*<0.01 against the CT group.

### Fecal short-chain fatty acid changes induced by the RE

**Figure 5:**
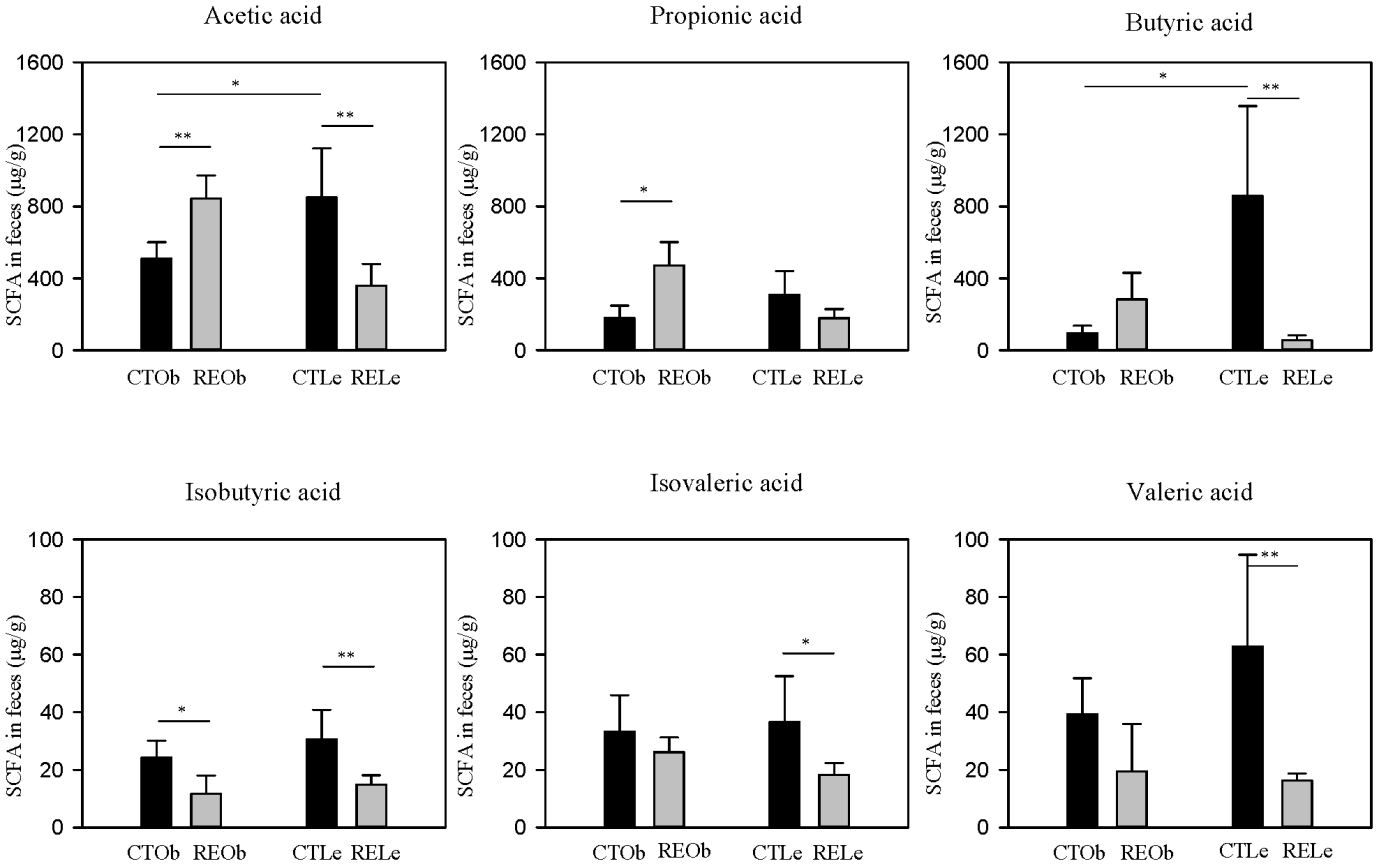
Effect of the RE-supplementation on SCFA fecal composition. Results are shown as µg/g of feces f.w. (fresh weight) in obese (Ob) and lean (Le) female Zucker rats fed a control (CT) diet or the diet supplemented with the rosemary extract (RE) rich in carnosic acid (CA, ∼40%). Significant differences between groups are indicated by *P<0.05, **P<0.01.

The concentration of total SCFA excreted in the feces was higher in the control lean Zucker rats than in their obese counterparts: 2145.6±640.6 and 878.5±72.4 µg/g (f.w.), respectively. This difference was still observed after the values were corrected by the daily fecal output (Table S3). The three major SCFA were acetic, propionic and butyric acid (∼90% of total) (Figure 5) and the ratios acetate/propionate were not significantly different between control Le and Ob rats (2.75 *vs*. 2.84, respectively). Valeric, isovaleric and isobutyric were present in lower and more similar quantities in the two genotypes. The consumption of the RE caused an increase in the three main SCFA in the obese rats whereas in the lean rats, the levels of the three SCFA were significantly reduced (Figure 5). These changes were maintained after the values were corrected by the daily fecal output (Table S3).The acetate/propionate ratio was slightly reduced in both types of animals (2.0 and 1.79 for the RE-supplemented lean and obese rats, respectively). The levels of isobutyric, valeric and isovaleric acids were all slightly diminished by the RE in both types of rats, most significantly in the lean rats.

## Discussion

In the present study we have shown for the first time that sustained consumption of a RE rich in the bioactive diterpenoid CA selectively modified the growth of a limited number of bacteria in the caecum: i) increased the *B. coccoides* and *Bacteroides*/*Prevotella* groups and reduced the *Lactobacillus*/*Leuconostoc*/*Pediococccus* group in lean and obese Zucker rats and, ii) increased the levels of the *Bifidobacterium* genus and reduced those of the *C. Leptum* group, most significantly, in the lean animals. In addition, the intake of the RE reduced caecum β-glucosidase activity (>90%) and increased the excretion of fecal fiber (7–8%) in the two types of animals. These results are concomitant with the body weight reducing effects and the metabolic and inflammatory beneficial properties of the RE [18], [19] and are suggestive of a potential prebiotic effect of the RE against metabolic disorders and obesity.

Since most investigated prebiotics are fibers, it is important to characterize and quantify the presence of fiber in bioactive enriched plant extracts. Inulin, fructo-oligosaccharides, cellulose, etc, are all prebiotics known to resist upper gut digestion and to reach the large intestine where they are fermented by the microbiota. The main site of bacterial fermentation in rats, the caecum, is enlarged after the intake of these fibers [28], [29]. The consumption of the RE caused a significant augmentation of the caecum weight suggesting the presence of additional non-digested fibers and carbohydrates in the extract. However, considering that the addition of the RE to the standard feed (0.5% w/w) did not modify quantitatively the composition of the main nutrients in the diet (Table S1) and that the RE did not affect the daily food intake [18], the contribution of the RE to the daily consumption of fiber and carbohydrates (as well as of the other nutrients) was minor (the total fiber and carbohydrates provided by the RE accounted for as little as ∼0.03% of the total fiber and 0.3% of the carbohydrates already present in the standard feed). Even if the fiber and carbohydrates from the RE had not been digested in the small intestine neither fermented in the caecum, they would not explain the increase in the fiber excreted in the feces of the animals that consumed the RE (∼7–8%). However, we cannot fully discard that some of the nutrients in the RE may have a part in the observed effects. It should be noted that the major difference between the two diets was the presence of diterpenoids which provided a daily intake of these compounds of ∼40 mg and ∼70 mg for the lean and obese rats, respectively [18]. We have previously shown that part of the ingested diterpenoids (CA, carnosol, CA 12-methyl ether) resist gastric and small intestine conditions and reach the large intestine [21] and therefore, it is plausible that these molecules and (or) their derived metabolites may be involved in the observed effects.

The evaluation of novel compounds or extracts with potential prebiotic effects should also take into account the effects of such products on digestive enzymes in the upper gut and in the hydrolysis and fermentation by the microbiota in the large bowel. We have previously reported that the intake of RE causes a significant inhibition of a butyrate-esterase activity in the stomach of Zucker rats and it was hypothesized that this may cause a reduction in the digestion and absorption of fat from the diet [18]. Fecal composition analysis in the present work does not support this hypothesis since we did not find significant differences in the % of fat eliminated in the feces between the control and RE-supplemented animals. We have now also evaluated the effects of the consumption of the RE on α-amylase activity in the small intestine and on β-glucosidase in the caecum of the rats. A number of intestinal bacteria exhibit β-glucosidase activity, including members of the saccharolytic Bifidobacteria, Lactobacilli and of the Clostridial cluster XIVa (*Eubacterium*, *Roseburia*) [15], [30]. Our results show that the intake of the RE did not affect the rat intestinal α-amylase activity. In contrast, the RE caused a substantial reduction of the β-glucosidase activity in the caecum of both the lean and obese animals suggesting that fermentation of non-digested polysaccharides such as cellulose might be substantially diminished. This was further supported by the significant increase in the fiber excreted in the feces of the supplemented animals. Fermentation of non-digested carbohydrates by saccharolytic bacteria is currently considered a beneficial effect for host metabolic health [15], [16], and thus inhibition of this process may be considered deleterious. However, fermentation in the caecum produces a significant daily quantity of SCFA which are partially assimilated into host carbohydrates and lipids equivalent to 8% to10% of caloric requirements [8], [9]. Inhibition of the enzyme activities involved in this process such as β-glucosidase may contribute to reduce dietary energy extraction and to moderate body weight gain which may be beneficial. β-Glucosidases are also de-glycosylating enzymes that can release a range of aglycones from plant compounds which may exhibit toxic or health-promoting activities [30]. The inhibition of this activity in the caecum by the RE can thus have an additional impact on health through regulation of bioactive microbial metabolites production.

It has been generally recognized that gut microbiota clearly differs between, genetically- or diet-induced, lean and obese phenotypes [3], [4], [31]. Genetic predisposition to obesity associated to leptin (*ob*/*ob* mice) or leptin receptor (*db*/*db* mice) deficiencies appear to shape a microbiota specialized for enhanced dietary energy recovery with elevated abundance of Firmicutes and reduced abundance of Bacteroidetes compared with lean mice [12], [32], [33]. A closer look at the specific enriched or depleted genus or species shows a significant variability depending on the representative sequence used in the analysis [33] and on other confounding factors (e.g. sex, age, diet composition, environmental factors, etc) [5]. Leptin receptor-deficient Zucker male obese rats [34] exhibited lower total bacteria but higher levels of Lab158 (most *Lactobacillus*, *Leuconostoc* and *Pediococcus* among other genus) and Erec482 (most members of *Clostridium* cluster XIVa) and no differences in Bac303 (most *Bacteroides* and *Prevotella*) compared to Zucker male lean rats. In our study, the levels of total bacteria were similar in the obese and lean female Zucker rats. We corroborated that the obese rats displayed elevated numbers of some members of the Firmicutes group, *Lactobacillus/Leuconostoc/Pediococcus* and *B. coccoides*, compared with their lean counterparts whereas the *C. leptum* and the Bacteroidetes groups were lower (although did not reach significance) in the obese rats. The saccharolytic *Bifidobacterium* genus, generally considered beneficial bacteria, has also been inversely associated with obesity [3] but while Waldram et al [34] confirmed lower levels of Bif164 (targeting most bifidobacteria) in the obese male Zucker rats, our results show the opposite reinforcing the potential effects of other variables in the microbiota (i.e. sex, diet, age, etc) and the difficulty of establishing definitive differences between obese and lean phenotypes. We also examined changes in some of the main caecum microbiota groups following the intake of the RE. The RE caused a moderate but significant reduction of total caecum bacteria. It has been reported that germ-free mice and antibiotic-treated mice display enlarged caecum and changes in the proportions of Firmicutes and Bacteroidetes proportions [35]–[37] which suggest that the RE might have a strain specific bactericidal and/or bacteriostatic effect in the caecum. Indeed, CA and carnosol exert some antimicrobial activities [38] and can be found in the caecum of the Zucker rats following the intake of the RE [21] and thus, we cannot discard a potential antibiotic effect of the RE in the gut.

Many prebiotics (fibers) can improve glucose and lipid homeostasis as well as the inflammatory status in the host and these properties have been associated with the growth of beneficial bacteria such as *Bifidobacterium* [4], [16], [31], [39]. Dietary supplementation with the probiotic *B. pseudocatenulatum* CECT 7765 can increase bifidobacteria in the gut and moderate serum lipids, insulin resistance, leptin and other inflammatory cytokines in high-fat fed mice [40]. In previous studies, we have shown that the consumption of the RE rich in CA downregulated serum triglycerides, cholesterol, insulin, leptin, TNF-α and Il-1β and upregulated adiponectin [18], [19] only in the lean Zucker rats revealing critical regulatory differences against the leptin-resistant obese rats. We now report that the consumption of RE is also linked to a different response in the caecum *Bifidobacterium* counts which are significantly increased in the lean animals and supports an association with the regulation of lipids and adipokines in this genotype. It should be noted that the numbers of *Bifidobacterium* exhibited a large variability in the obese rats supplemented with the RE. Although all the qPCR analyses were carried out following the same protocols, under the same conditions and at the same time, we cannot fully discard the possibility of some qPCR inhibitors in this group. However, the response of the obese animals may be more variable since the obesity has been reported to worsen or slow the capacity of these animals to respond to nutritional or pharmacological treatment and thus, it may be more difficult to exert a regulatory effect in these animals [41]. In addition, we found that the *C. leptum* group was also differentially regulated between the obese and lean genotype in response to the RE. Principal component analysis of the current results for bacterial groups and the previously reported serum biochemical variables [18], [19] (Figure 3) showed that these two genotypes could be easily distinguished from each other and that the lean rats responded to the RE more prominently than the obese ones. Overall, our data support a host genetic effect in the gut microbiota present in the lean and obese animals and that each type of rat could have specific metabolic and inflammatory status as well as different responses to dietary compounds linked to their specific microbiomes [34].

Prebiotic fibers that promote fermentation lead, in general, to higher levels of caecum and fecal SCFA [42]. These metabolites act as mediators between gut microbiota and host inflammatory status since they can function as signaling molecules suppressing the production of inflammatory cytokines [43], [44], regulating the production of gut hormones such as glucagon-like peptide-1 (GLP-1) and, consequently, insulin secretion [45] or inducing the production of leptin [46]. The acetate/propionate ratio is also critical in regulating cholesterol, lipids and glucose synthesis in the host [47]. To determine whether the effects of the RE in the caecum had an impact on the levels of SCFA, we analyzed the concentration of these metabolites in feces. We found significant differences between control lean and obese rats and between control and RE-supplemented animals suggesting important differences in the production, absorption and metabolism of these metabolites between the two types of rats. Microbiota and SCFA metabolism differences between the lean and obese Zucker rats may be essential to explain the different metabolic and inflammatory response of the animals to the RE and warrant further research.

Other plant extracts rich in bioactive compounds (e.g. polyphenols), polyphenols themselves and/or their derived metabolites have been shown to confer some of the effects attributed to prebiotics [48]. A pomegranate extract, rich in punicalagin and ellagic acid, increases caecum content and *Bifidobacterium* and reduces serum cholesterol and the expression of inflammatory markers in mice adipose tissue [49] and, both a pomegranate extract and its main microbiota-derived metabolite urolithin increase the counts of fecal *Bifidobacterium*, *Lactobacillus* and *Clostridium* spp. and decrease inflammatory markers in the rat [50]. Wild blueberry [51], green tea extracts [52] and resveratrol [53] are all able to increase counts of *Lactobacillus* and/or *Bifidobacterium*. Although some of these plant products need to be fully characterized to determine or exclude the presence of prebiotic fiber, these results and our results show that plant bioactive compounds from different origins and molecular composition or their derived metabolites are able to modify gut microbiota composition and to promote beneficial changes with an impact into the host metabolism and inflammatory response. It is conceivable that plant food bioactive compounds may gradually be considered or classified as ‘prebiotics’ or as compounds with some ‘prebiotic effects’. The latest prebiotic concept [15], [16] may need to be expanded in the future to include these other food components, even though they are not necessarily fermented by the microbiota. Bioactive-enriched plant derived products constitute an additional strategy to combat metabolic disorders and associated inflammatory processes but further research is required to fully characterize these products, to unravel their mechanisms of action and to demonstrate their effects in humans.

In conclusion, a RE enriched in bioactive diterpenoids (mostly, CA) and that exhibits body weight reducing effects and beneficial metabolic and inflammatory properties [18], [19] has also a significant impact on the microbiota composition and β-glucosidase activity in the caecum of female Zucker rats and increases fiber fecal excretion. Importantly, the presence of a recessive mutation in the leptin receptor (*fa/fa*, obese rats) has a critical effect on the microbiota caecum composition and on the host response to the consumption of RE. A limitation of our study is that only a few groups of bacteria members of the Firmicutes, Bacteroidetes, and Actinobacteria phyla were investigated and that they constitute a small % of the total bacteria. With the application of more potent technologies (metagenomics) and the identification of new species of bacteria, we shall progressively know and understand better the gut microbiota, its modulation by the diet and its relationship with the host health status.

## Supporting Information

Figure S1 **Relative food intake (RFI) for lean (Le) and obese (Ob) female Zucker rats fed a control diet (CT) or a rosemary extract (RE)-supplemented diet.**
(TIFF)

Figure S2 **Weekly distribution of fecal weight (g/rat day) and stools volume (mm^3^) at the end of the study (insert) for CT and RE-supplemented obese (Ob) and CT and RE-supplemented lean (Le) female Zucker rats.** Significant differences at each time point are indicated by *P<0.05, **P<0.01, ***P<0.001.
(TIFF)

Table S1 **Effect of the addition of RE on the nutrient composition of the standard diet (CT).**
(DOCX)

Table S2 **Primers and probes used for the quantification of bacteria in the cecum content samples using Q-PCR assays targeting 16S rRNA coding regions.**
(DOCX)

Table S3 **SCFA composition of feces from lean (Le) and obese (Ob) female Zucker rats following the intake of a control diet (CT) or the same diet supplemented with the rosemary extract (RE, 0.5% w/w) enriched in carnosic acid (CA, ∼40%).**
(DOCX)
