# Supplementary figures and images for: A Rosemary Extract Rich in Carnosic Acid Selectively Modulates Caecum Microbiota and Inhibits β-Glucosidase Activity, Altering Fiber and Short Chain Fatty Acids Fecal Excretion in Lean and Obese Female Rats
Source: PLoS One. 2014 Apr 14;9(4):e94687. doi: 10.1371/journal.pone.0094687 (PMC3986085; doi:10.1371/journal.pone.0094687)

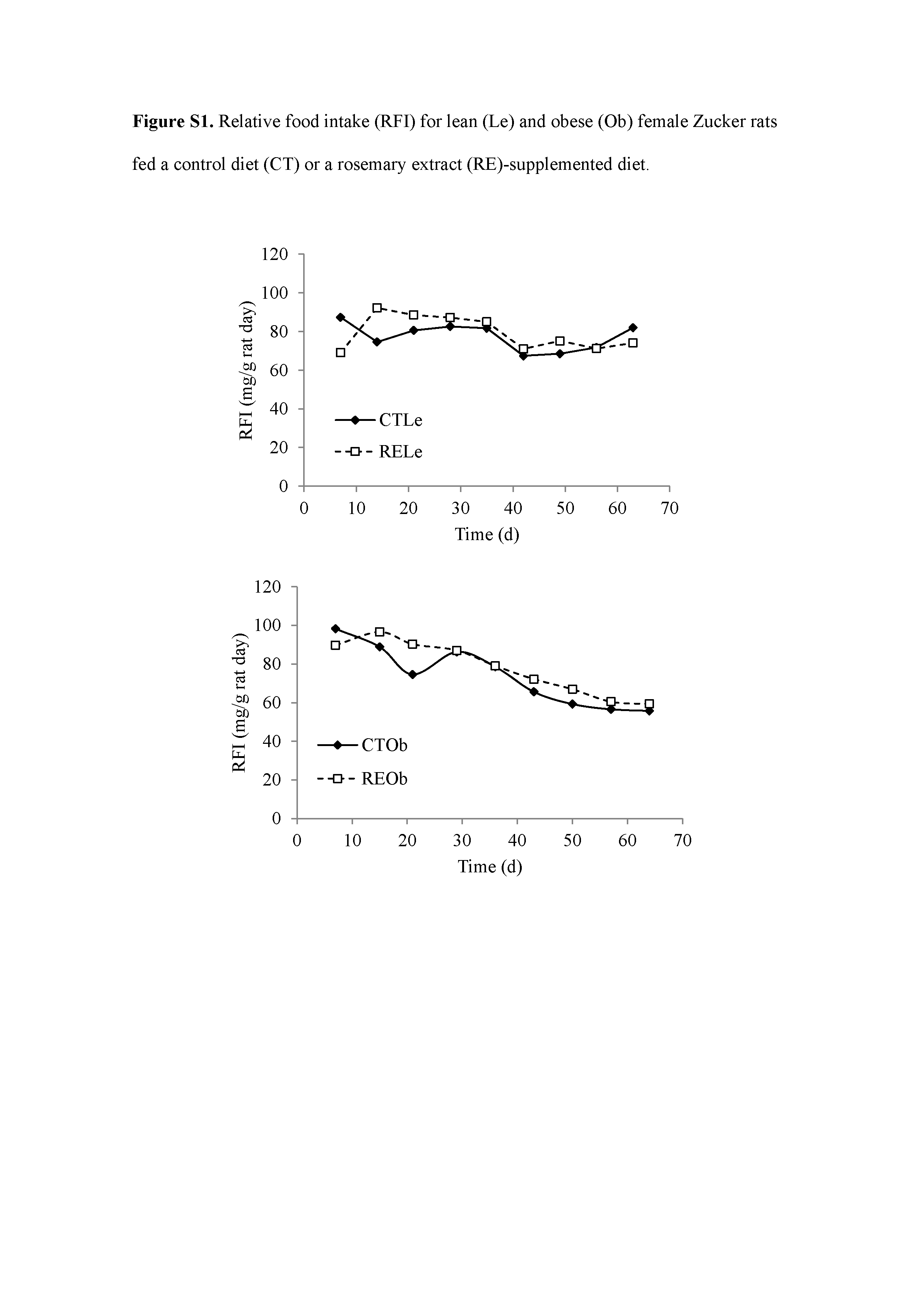

Supplement: Figure S1 — Relative food intake (RFI) for lean (Le) and obese (Ob) female Zucker rats fed a control diet (CT) or a rosemary extract (RE)-supplemented diet. (TIFF) [file pone.0094687.s001.tiff]

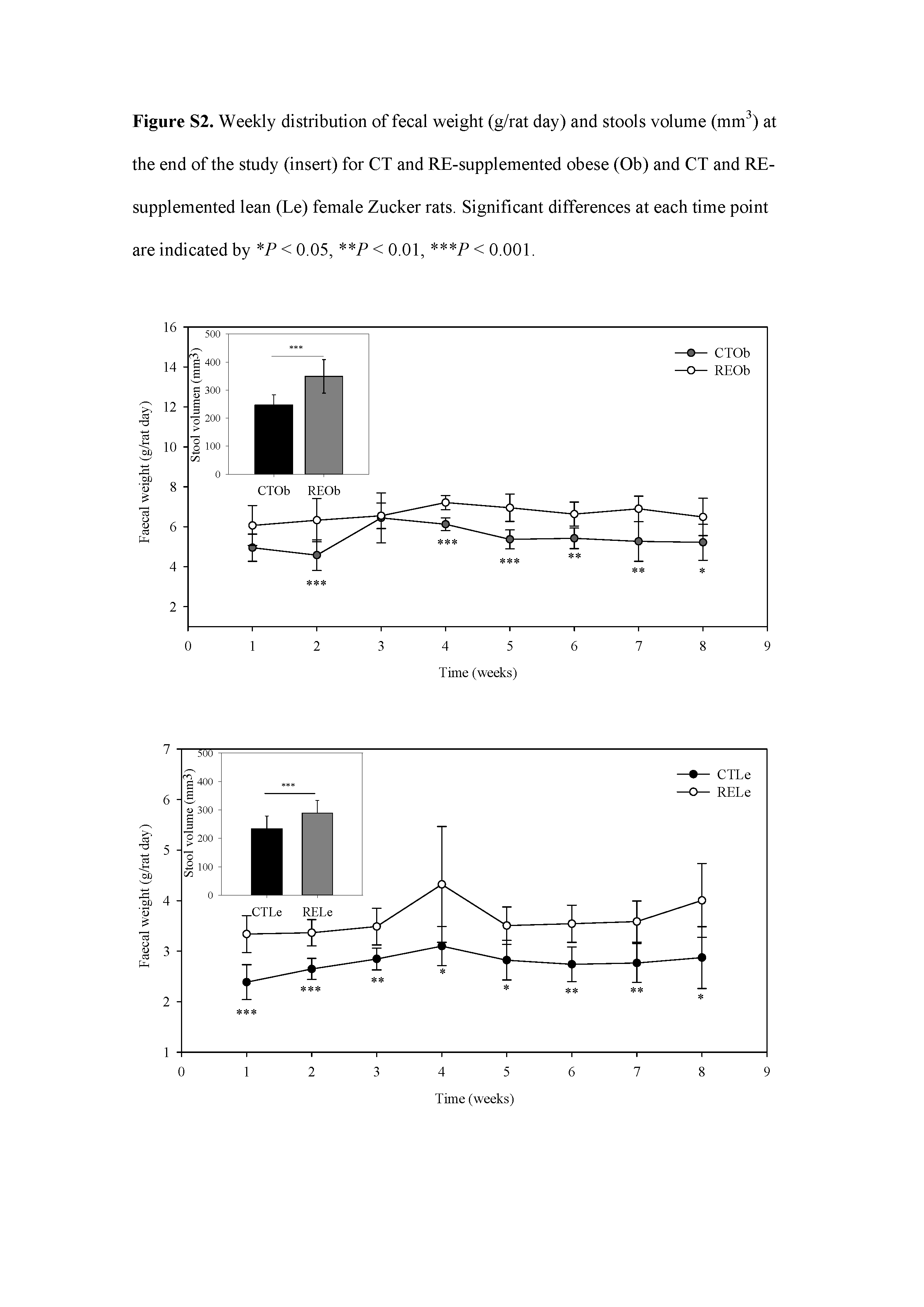

Supplement: Figure S2 — Weekly distribution of fecal weight (g/rat day) and stools volume (mm3) at the end of the study (insert) for CT and RE-supplemented obese (Ob) and CT and RE-supplemented lean (Le) female Zucker rats. Significant differences at each time point are indicated by *P<0.05, **P<0.01, ***P<0.001. (TIFF) [file pone.0094687.s002.tiff]
