# Supplementary material for: A Rosemary Extract Rich in Carnosic Acid Selectively Modulates Caecum Microbiota and Inhibits β-Glucosidase Activity, Altering Fiber and Short Chain Fatty Acids Fecal Excretion in Lean and Obese Female Rats
Source: PLoS One. 2014 Apr 14;9(4):e94687. doi: 10.1371/journal.pone.0094687 (PMC3986085; doi:10.1371/journal.pone.0094687)
Supplement: Table S1 — Effect of the addition of RE on the nutrient composition of the standard diet (CT). (DOCX) [file pone.0094687.s003.docx]

**Table** S**1**. Effect of the addition of RE on the nutrient composition of the standard diet (CT).

| Major nutrients | Control (CT) Diet^1^ (g/100 g) | RE^2^-supplemented Diet (5 g RE /Kg diet) (g/100 g) |
| --- | --- | --- |
| Protein | 14.30 | 14.23 |
| Fat | 4.00 | 4.02 |
| Carbohydrates | 48.00 | 47.89 |
| Fiber^3^ | 22.10 | 22.00 |
| Ash | 4.70 | 4.71 |
| CA and other diterpens | - | 0.27 |
| Water | 6.90 | 6.88 |
| Energy (Kcal/g) | 2.90 | 2.91 |

|  |
| --- |

^1^ Harlan, Teklad Diets, Madison WI; ^2^ Rosemary extract composition: protein 0.6%, fat 7.9%, fiber 1.0%, carbohydrates 29.1%, ashes 6.2%, CA+ other diterpens ~53%, water 2.2%, energy 4.04 Kcal/g; ^3^ Total dietary fiber.
