# Supplementary material for: A Rosemary Extract Rich in Carnosic Acid Selectively Modulates Caecum Microbiota and Inhibits β-Glucosidase Activity, Altering Fiber and Short Chain Fatty Acids Fecal Excretion in Lean and Obese Female Rats
Source: PLoS One. 2014 Apr 14;9(4):e94687. doi: 10.1371/journal.pone.0094687 (PMC3986085; doi:10.1371/journal.pone.0094687)
Supplement: Table S2 — Primers and probes used for the quantification of bacteria in the cecum content samples using Q-PCR assays targeting 16S rRNA coding regions. (DOCX) [file pone.0094687.s004.docx]

**Table S2.** Primers and probes used for the quantification of bacteria in the cecum content samples using qPCR assays targeting 16S rRNA coding regions.

|  |  |  |  |  |
| --- | --- | --- | --- | --- |
| Target organism | Primers and  Probes | Sequence (5´-3´) | nM | Reference |
| *Lactobacillus/Leuconostoc/*  *Pediococcus* group | F_Lacto 05 | AGC AGT AGG GAA TCT TCC A | 300 | [23] |
|  | R_Lacto 04 | CGC CAC TGG TGT TCY TCC ATA TA | 300 |  |
| *Blautia coccoides* group | F_Ccoc 07 | GAC GCC GCG TGA AGG A | 300 | [23] |
|  | R_Ccoc 14 | AGC CCC AGC CTT TCA CAT C | 300 |  |
|  | P_Erec482* | VIC-CGG TAC CTG ACT AAG AAG | 250 | [25] |
| *Bacteroides/Prevotella* group | F_Bacter 11 | CCT WCG ATG GAT AGG GGT T | 400 | [23] |
|  | R_Bacter 08 | CAC GCT ACT TGG CTG GTT CAG | 400 |  |
|  | P_Bac303* | VIC-AAG GTC CCC CAC ATT G | 250 | [24] |
| *Clostridium leptum* group | F_Clept 09 | CCT TCC GTG CCG SAG TTA | 300 | [23] |
|  | R_Clept 08 | GAA TTA AAC CAC ATA CTC CAC TGC TT | 300 |  |
|  | P-Clep 01* | 6FAM-CAC AAT AAG TAA TCC ACC | 250 |  |
| *Bifidobacterium* genus | F_Bifid09c  R_Bifid06  P_Bifid* | CGG GTG AGT AAT GCG TGA CC  TGA TAG GAC GCG ACC CCA  6FAM-CTC CTG GAA ACG GGT G | 300  300  250 | [23] |
| *Total bacteria* | PRBA338f  P518r | ACT CCT ACG GGA GGC AGC AG  ATT ACC GCG GCT GCT GG | 300  300 | [22] |

* Taqman probes designed with Molecular-Groove Binding Non-fluorescence Quencher.
