# Supplementary material for: A Rosemary Extract Rich in Carnosic Acid Selectively Modulates Caecum Microbiota and Inhibits β-Glucosidase Activity, Altering Fiber and Short Chain Fatty Acids Fecal Excretion in Lean and Obese Female Rats
Source: PLoS One. 2014 Apr 14;9(4):e94687. doi: 10.1371/journal.pone.0094687 (PMC3986085; doi:10.1371/journal.pone.0094687)
Supplement: Table S3 — SCFA composition of feces from lean (Le) and obese (Ob) female Zucker rats following the intake of a control diet (CT) or the same diet supplemented with the rosemary extract (RE, 0.5% w/w) enriched in carnosic acid (CA, ∼40%). (DOCX) [file pone.0094687.s005.docx]

**Table S3.** SCFA composition of feces from lean (Le) and obese (Ob) female Zucker rats following the intake of a control diet (CT) or the same diet supplemented with the rosemary extract (RE, 0.5 % w/w) enriched in carnosic acid (CA, ~40%).

| SCFA | CTOb | | REOb | | CTLe | | RELe | |
| --- | --- | --- | --- | --- | --- | --- | --- | --- |
|  | μg/g | mg/rat day^1^ | μg/g | mg/rat day | μg/g | mg/rat day | μg/g | mg/rat day |
| Acetate | 508.1 ± 92.6 | 2.8 | 844.9 ± 127.4 | 5.6 | 849.4 ± 273.5 | 2.4 | 358.3 ± 122.0 | 1.3 |
| Propionate | 178.8 ± 69.9 | 1.0 | 471.9 ± 129.1 | 3.1 | 309.0 ± 131.1 | 0.9 | 178.7 ± 50.8 | 0.7 |
| Butyrate | 94.8 ± 42.5 | 0.5 | 280.6 ± 147.8 | 1.9 | 857.4 ± 501.4 | 2.4 | 56.6 ± 26.3 | 0.2 |
| Isobutyrate | 24.2 ± 6.0 | 0.1 | 11.7 ± 6.3 | 0.1 | 30.5 ± 10.4 | 0.1 | 14.8 ± 3.3 | 0.05 |
| Isovalerate | 33.4 ± 12.6 | 0.2 | 26.2 ± 5.1 | 0.2 | 36.6 ± 16.0 | 0.1 | 18.3 ± 4.0 | 0.1 |
| Valerate | 39.3 ± 12.6 | 0.2 | 19.4 ± 16.6 | 0.1 | 62.8 ± 32.0 | 0.2 | 16.3 ± 2.5 | 0.1 |
| TOTAL | 878.5 ± 72.4 | 4.8 | 1654.7 ± 413.6 | 11.0 | 2145.6 ± 640.6 | 6.1 | 643.1 ± 179.8 | 2.5 |

^1^: multiplied by the average fecal output (CTOb: 5.43, REOb: 6.64, CTLe: 2.77, RELe: 3.64 g feces/rat day)
